# Linear Extended State Observer-Based Motion Synchronization Control for Hybrid Actuation System of More Electric Aircraft

**DOI:** 10.3390/s17112444

**Published:** 2017-10-25

**Authors:** Xingjian Wang, Rui Liao, Cun Shi, Shaoping Wang

**Affiliations:** School of Automation Science and Electrical Engineering, Beihang University, Beijing 100191, China; liaorui@buaa.edu.cn (R.L.); shicun@buaa.edu.cn (C.S.); shaopingwang@vip.sina.com (S.W.)

**Keywords:** hybrid actuation system, electro-hydraulic servo actuator, electromechanical actuator, more electric aircraft, motion synchronization control, extended state observer

## Abstract

Moving towards the more electric aircraft (MEA), a hybrid actuator configuration provides an opportunity to introduce electromechanical actuator (EMA) into primary flight control. In the hybrid actuation system (HAS), an electro-hydraulic servo actuator (EHSA) and an EMA operate on the same control surface. In order to solve force fighting problem in HAS, this paper proposes a novel linear extended state observer (LESO)-based motion synchronization control method. To cope with the problem of unavailability of the state signals required by the motion synchronization controller, LESO is designed for EHSA and EMA to observe the state variables. Based on the observed states of LESO, motion synchronization controllers could enable EHSA and EMA to simultaneously track the desired motion trajectories. Additionally, nonlinearities, uncertainties and unknown disturbances as well as the coupling term between EHSA and EMA can be estimated and compensated by using the extended state of the proposed LESO. Finally, comparative simulation results indicate that the proposed LESO-based motion synchronization controller could reduce significant force fighting between EHSA and EMA.

## 1. Introduction

Actuation system is a key factor in the aircraft, which drives the control surface to manipulate the aircraft’s attitude and flight path [1]. For a long time, the actuation system is merely driven by hydraulic power. However, as the aircraft industry thriving, an exuberant demand for safety and reliability in airplane has motivated significant adoption of the similar redundant hydraulic actuators. However, the common-mode/common-cause (CM/CC) fault would be the potential risk in the similarly redundant actuation system composed of two hybrid actuation systems (HAS), which limits further improvement to the reliability of such actuation systems [2,3].

Moving towards the more electric aircraft (MEA), a hybrid actuator configuration provides an opportunity to introduce an electromechanical actuator (EMA) into primary flight control [4,5]. Therefore, applying dissimilar redundant hybrid actuation system (HAS), which consists of an electro-hydraulic servo actuator (EHSA) and an electromechanical actuator (EMA), is considered as an improvement in reliability for advanced aircraft design [6]. The “more electric” focus will permit us to reduce the number of power transfer system functions and utilize the potential of ultra-reliable miniaturized power electronics, fault-tolerant electrical distribution systems and electric generator/motor drives/actuators to increase performance, and reduce the costs [7,8]. Thus, HAS composes of an EHSA and an EMA could combine the merits of both that will effectively avoid the CM/CC faults and improve the robustness of the actuation system [9].

Adopting the HAS in aircraft also introduces some problems that need to be addressed. It is obvious that EHSA and EMA are of different operating principles, causing different dynamic responses with the same input signal [10]. When bounding those two actuators as a whole to drive the control surface through rigid coupling, intercoupling effect between the outputs of EHSA and EMA would appear. All these issues result in a serious force fighting problem when these two actuators are operated together to drive the control surface, which may affect the accuracy of the tracking control or even damage the control surface [2]. Therefore, solving the non-synchronous outputs of two different actuators is a major issue in developing the controller for HAS in modern aviation industry.

In order to achieve the synchronous force outputs of two different actuators, the difference between the average actuator forces and the actual actuator force was introduced to an integrator to generate a position demand offset to eliminate force fighting [11]. The researches [10,12] also illustrate the static force fighting reduction controller designing for a hybrid actuation system consisting of a EHSA and EMA. However, in pursuit of further enhancement in the tracking control performance, the nonlinear dynamics, uncertainties, and the external disturbances, as well as the coupling effect between EHSA and EMA, should be fully taken into consideration while designing the controller.

An effective solution of the force fighting issue between the two different actuators is to introduce the motion state synchronization method. The motion states of those actuators including displacement, velocity, acceleration, jerk and etc. can be maintained in a consistent condition by adopting motion state synchronization [2]. Acquiring these state variables in HAS could enable the estimation of the system movement. Cochoy et al. designed a force equalization controller using the state signals of displacement, velocity and force [4], and the controller was synthesized based on the ideal hypothesis that all signals involved are available. Nevertheless, it is not easy to get all these state signals of the actual actuation system on aircraft. To cope with this issue, the hybrid actuation system test benches was built to obtain all state signals by adding multiple sensors [6]. However, these methods would greatly increase the cost and weight of the actuators, which greatly limits its application on the aircraft.

The main contribution of this study is to propose a novel linear extended state observer (LESO)-based motion synchronization control method without increasing any additional sensor. In terms of acquiring the requisite state variables without additional sensors, constructing a suitable state observer is a feasible method. Therefore, the LESO is designed to estimate the state variables including the nonlinear dynamics, uncertainties, and the external disturbances, as well as the coupling term between EHSA and EMA. The difficult issue of obtaining the state signals for motion synchronization controller can be solved by the proposed observer without increasing any sensor. Then the synchronization controllers will be developed according to the observed states to compensate those unknown disturbances and the coupling force by appropriately reassigning the control signals to the actuators. The dynamic force fighting between EHSA and EMA is supposed to be reduced by the proposed ESO-based motion synchronization control method and satisfactory tracking control performance can be achieved by applying the proposed controller.

## 2. Dynamic Models and Problem Formulation

**Figure 1:**
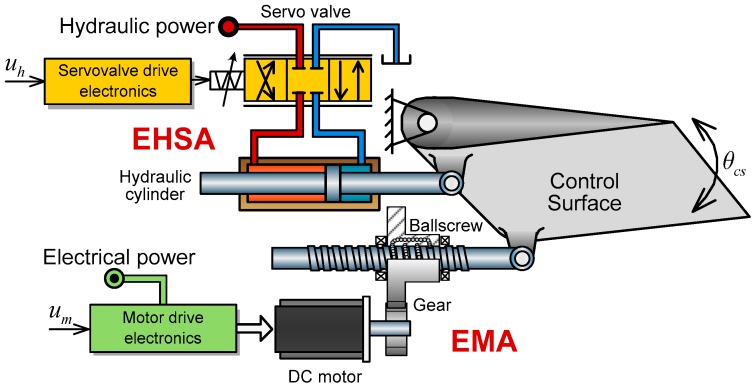
The structure diagram of hybrid actuation system.

The structure diagram of HAS composed by EHSA and EMA is shown in Figure 1.

In thus a hybrid system, EHSA and EMA are controlled by flight control input signals $u_{h}$ and $u_{m}$ respectively, to drive the control surface of aircraft. EHSA is a typical servo-valve controlled hydraulic position control system while EMA is an electromechanical position control system composed by electric motor, gear, and ballscrew.

### 2.1. The Dynamic Model of Control Surface in HAS

As shown in Figure 1, the dynamics of the control surface can be given as:

(1) $$(F_{h}+F_{m})r_{cs}=J_{cs}{\ddot{\theta }}_{cs}+F_{air}r_{cs}$$

(2) $$F_{h}=K_{h}(x_{h}-x_{cs})$$

(3) $$F_{m}=K_{m}(x_{m}-x_{cs})$$

where $J_{cs}$ and $\theta_{cs}$ is equivalent moment of inertia and angular displacement of the control surface respectively, $r_{cs}$ is the radial distance of the control surface, $F_{air}$ is the external air disturbance acting on the control surface. $F_{h}$ and $F_{m}$ are the output forces of EHSA and EMA, and $K_{h}$ and $K_{e}$ are the connection stiffness of EHSA and EMA, respectively.

Usually, the variation range of angular displacement $\theta_{cs}$ falls into −0.35 rad to +0.35 rad, therefore, the relationship between $\theta_{cs}$ and $x_{cs}$ can be approximately considered as linear [13] and can be described ad:

(4) $$x_{cs}=\theta_{cs}r_{cs}$$

### 2.2. The Dynamic Model of EHSA

The conventional EHSA typically consists of a servo valve, a symmetrical hydraulic cylinder and other accessories. According to previous studies [14,15,16], the servo valve can be described by a proportional function as:

(5) $$x_{v}=K_{v}u_{h}+\delta_{v}$$

where $x_{v}$ is the spool valve displacement, $K_{v}$ is the amplification coefficient and $\delta_{v}$ is the unmodeled dynamics of servo valve.

The input flow of hydraulic cylinder can be given as:

(6) $$Q_{h}=K_{q}K_{v}u_{h}-K_{c}P_{h}+K_{q}\delta_{v}+\delta_{q}$$

where $K_{q}$ is the flow/opening gain and $K_{c}$ is the flow/pressure gain, $P_{h}$ is the load pressure of the hydraulic cylinder, $\delta_{q}$ represents the effect of unmodeled dynamics and uncertainties.

Then, the flow dynamics and force dynamics of EHSA can be described as:

(7) $$\{\begin{array}{ccc} Q_{h} & = & A_{h}{\dot{x}}_{h}+\frac{V_{h}}{4E_{h}}{\dot{P}}_{h}+C_{hl}P_{h}\\ A_{h}P_{h} & = & m_{h}{\ddot{x}}_{h}+B_{h}{\dot{x}}_{h}+F_{h}+d_{h} \end{array}$$

where $A_{h}$ is the piston area, $V_{h}$ is the volume of the piston chamber, $E_{h}$ is the effective bulk modulus, $C_{hl}$ is the total leakage coefficient of the cylinder, $m_{h}$ is the mass of piston and rod, $B_{h}$ is the damping coefficient of the cylinder, and $d_{h}$ represents unknown external disturbances.

Define $x_{1}={\left[x_{11},\;x_{12},\;x_{13}\right]}^{T}={\left[x_{h},\;{\dot{x}}_{h},\;{\ddot{x}}_{h}\right]}^{T}$ as the state vector of EHSA and let $u_{1}=u_{h}$, the state-space form of EHSA system can be given as:

(8) $$\Psi_{\mathrm{EHSA}}：\{\begin{array}{c} {\dot{x}}_{11}=x_{12}\\ {\dot{x}}_{12}=x_{13}\\ {\dot{x}}_{13}=f_{1}(x_{1})+g_{1}+\varphi_{1}+b_{1}u_{1} \end{array}$$

where $f_{1}(x_{1})=-\frac{4E_{h}K_{hs}(K_{h}+C_{hl})}{m_{h}V_{h}}x_{11}-\frac{4E_{h}A_{h}^{2}+4E_{h}B_{h}(K_{h}+C_{hl})+V_{h}}{m_{h}V_{h}}x_{12}-\frac{4E_{h}m_{h}(K_{h}+C_{hl})+B_{h}V_{h}}{m_{h}V_{h}}x_{13}$, $g_{1}=\frac{4E_{h}K_{hs}(K_{h}+C_{hl})}{m_{h}V_{h}}x_{cs}-\frac{K_{hs}}{m_{h}}{\dot{x}}_{cs}$, $\varphi_{1}=-\frac{4A_{h}E_{h}K_{q}}{m_{h}V_{h}}\delta_{v}-\frac{4A_{h}E_{h}}{m_{h}V_{h}}\delta_{q}-\frac{4E_{h}(K_{h}+C_{hl})}{m_{h}V_{h}}d_{h}-\frac{1}{m_{h}}{\dot{d}}_{h}$ $b_{1}=$ $\frac{4A_{h}E_{h}K_{q}K_{v}}{m_{h}V_{h}}$.

Here, $g_{1}$ represents the coupling effect between EHSA and EMA, which is transferred by the control surface, $\varphi_{1}$ is the lumped effect from unmodeled dynamics, model uncertainties and unknown external disturbances, $b_{1}$ is the input gain.

### 2.3. The Dynamic Model of EMA

A typical EMA, shown in Figure 1, consists of a brushless DC motor, a gear box, a ballscrew actuator and other accessories [6,17]. The electrical dynamics of the brushless DC motor can be given as:

(9) $$\{\begin{array}{c} u_{m}=K_{e}\omega_{m}+L_{e}\frac{di_{e}}{dt}+R_{e}i_{e}\\ T_{em}=K_{em}i_{e} \end{array}$$

where $\omega_{m}$ is the angular velocity of motor rotator, $i_{e}$ is the current of the motor, $L_{e}$ and $R_{e}$ are the inductance and resistance of motor, respectively, $K_{em}$. back-EMF coefficient and $K_{em}$ is electromagnetic coefficient, $T_{em}$ is electromagnetic torque.

The mechanical dynamics of the EMA can be described as a lumped mass model, all rotating parts of the reduction gear, the ballscrew nut as well as the connection shafts are represented by the following equation:

(10) $$T_{em}=J_{m}\frac{d\omega_{m}}{dt}+B_{m}\omega_{m}+T_{l}+\delta_{gs}$$

where $J_{m}$ is the total moment of inertia of all rotating parts of EMA, $B_{m}$ the damping coefficient of EMA, $T_{l}$ is the output torque of the motor, $\delta_{gs}$ represents the uncertainty in the gear box.

The transition relationship between the rotational part and the translational part can be described as:

(11) $$\{\begin{array}{l} F_{m}=T_{l}K_{gs}\eta_{s}-d_{m}\\ \omega_{m}={\dot{x}}_{m}K_{gs}\eta_{s} \end{array}$$

where $K_{gs}$ and $\eta_{s}$ are the transmission coefficient and the transmission efficiency of the gear box and the ballscrew, respectively. $d_{m}$ represents disturbance force.

Define $x_{2}={\left[x_{21},\;x_{22},\;x_{23}\right]}^{T}={\left[x_{m},\;{\dot{x}}_{m},\;{\ddot{x}}_{m}\right]}^{T}$ as the state vector of EMA servo system, and let $u_{2}=u_{m}$ as the input of EMA system, then the state-space form of EMA can be given as:

(12) $$\Psi_{\mathrm{EMA}}：\{\begin{array}{c} {\dot{x}}_{21}=x_{22}\\ {\dot{x}}_{22}=x_{23}\\ {\dot{x}}_{23}=f_{2}(x_{2})+g_{2}+\varphi_{2}+b_{2}u_{2} \end{array}$$

where $f_{2}(x_{2})=-\frac{R_{e}K_{ms}}{L_{e}J_{m}K_{gs}^{2}\eta_{s}^{2}}x_{21}-\frac{K_{em}K_{e}K_{gs}^{2}\eta_{s}^{2}+L_{e}K_{ms}+R_{e}B_{m}K_{gs}^{2}\eta_{s}^{2}}{L_{e}J_{m}K_{gs}^{2}\eta_{s}^{2}}x_{22}-\frac{B_{m}L_{e}+J_{m}R_{e}}{J_{m}L_{e}}x_{23}$, $g_{2}=\frac{R_{e}K_{ms}}{L_{e}J_{m}K_{gs}^{2}\eta_{s}^{2}}x_{cs}$ $+\frac{K_{ms}}{J_{m}K_{gs}^{2}\eta_{s}^{2}}{\dot{x}}_{cs}$, $\varphi_{2}=\frac{R_{e}}{L_{e}J_{m}K_{gs}\eta_{s}}\delta_{gs}+\frac{1}{J_{m}K_{gs}\eta_{s}}{\dot{\delta }}_{gs}-\frac{R_{e}}{L_{e}J_{m}K_{gs}^{2}\eta_{s}^{2}}d_{m}-\frac{1}{J_{m}K_{gs}^{2}\eta_{s}^{2}}{\dot{d}}_{m}$, $b_{2}=\frac{K_{em}}{L_{e}J_{m}K_{gs}\eta_{s}}$. Here, $g_{2}$ represents the coupling effect between EHSA and EMA, which is transferred by the control surface, $\varphi_{2}$ is the lumped effect from unmodeled dynamics, model uncertainties and unknown external disturbances, and $b_{2}$ is the input gain of the EMA system.

### 2.4. Problem Formulation

Considering that the control surface is almost rigid, it is reasonable to neglect the dynamics of the control surface. Therefore, ensuring the synchronic outputs of EHSA and EMA is a significant part to maintain the displacement of the control surface $x_{cs}$ tracks the input command $x_{cmd}$. In another word, we need to design a controller to make the outputs of EHSA and EMA track the same desired trajectory $x_{cmd}$ at the same time and to achieve the minimum force fighting between the EHSA and EMA.

The desired motion trajectories are set by the trajectory generator [18] which is a second-order transfer function given as:

(13) $$x_{tr}=\frac{\omega_{tr}^{2}}{s^{2}+2\xi_{tr}\omega_{tr}s+\omega_{tr}^{2}}x_{cmd}$$

where the reference damping factor $\xi_{tr}$ and the reference frequency $\omega_{tr}$ are two design parameters of the trajectory generator.

Define the reference trajectory vector as $x_{r}={\left[x_{tr},\;{\dot{x}}_{tr},\;{\ddot{x}}_{tr}\right]}^{T}$ where the reference position $x_{tr}$, reference velocity ${\dot{x}}_{tr}$, and reference acceleration ${\ddot{x}}_{tr}$ are three output of the trajectory generator Equation (13). In the following section, we will design a motion synchronization controller to make the motion states of EHSA and EMA tracking the reference trajectory vector $x_{r}$.

## 3. LESO-based Motion Synchronization Controller Design

**Figure 2:**
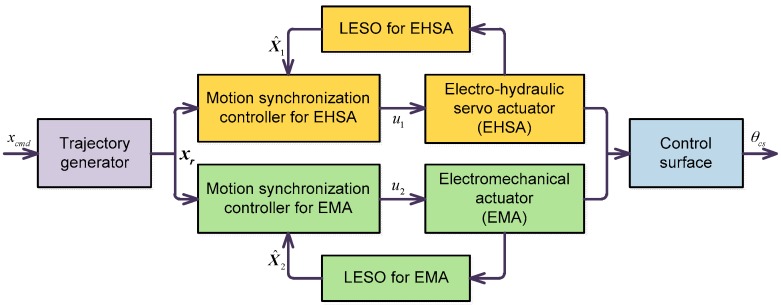
The overall schematic diagram of linear extended state observer (LESO)-based motion synchronization controller.

The overall schematic diagram of the proposed LESO-based motion synchronization controller is shown in Figure 2. The proposed control scheme consists of a trajectory generator which has been given in Section 2, an LESO for EHSA and an LESO for EMA, a synchronization controller for EHSA and a synchronization controller for EMA.

### 3.1. Design State Feedback Linearization Controller

As stated earlier, the main aim of this work is to eliminate force fighting in HAS by designing a motion synchronization controller based on motion states of EHSA/EMA. Therefore, a state feedback linearization controller is essential to achieve the expected motion state synchronization. Indeed, it has been shown that the state space models $\Psi_{\mathrm{EHSA}}$ and $\Psi_{\mathrm{EMA}}$ of EHSA and EMA are globally linearizable by nonlinear static state feedback.

To this end, the following state feedback linearization controller is presented as:

(14) $$u_{1}=\frac{1}{b_{1}}\left[-f_{1}(x_{1})-g_{1}-\varphi_{1}+v_{1}\right]$$

(15) $$u_{2}=\frac{1}{b_{2}}\left[-f_{2}(x_{2})-g_{2}-\varphi_{2}+v_{2}\right]$$

Applying the control laws Equations (14) and (15) to $\Psi_{\mathrm{EHSA}}$ and $\Psi_{\mathrm{EMA}}$ results into the following linear relationship between the states and new inputs $v_{1}$ and $v_{2}$:

(16) $$\Psi_{\mathrm{EHSA}}\Rightarrow \{\begin{array}{c} {\dot{x}}_{11}=x_{12}\\ {\dot{x}}_{12}=x_{13}\\ {\dot{x}}_{13}=v_{1}\\ y_{1}=x_{11} \end{array}$$

(17) $$\Psi_{\mathrm{EMA}}\Rightarrow \{\begin{array}{c} {\dot{x}}_{21}=x_{22}\\ {\dot{x}}_{22}=x_{23}\\ {\dot{x}}_{23}=v_{2}\\ y_{2}=x_{21} \end{array}$$

Now, we can design the new inputs $v_{1}$ and $v_{2}$ as:

(18) $$v_{1}={\dot{x}}_{r3}+k_{11}(x_{r1}-x_{11})+k_{12}(x_{r2}-x_{12})+k_{13}(x_{r3}-x_{13})$$

(19) $$v_{2}={\dot{x}}_{r3}+k_{21}(x_{r1}-x_{21})+k_{22}(x_{r2}-x_{22})+k_{23}(x_{r3}-x_{23})$$

where $x_{r1}=x_{tr}$, $x_{r2}={\dot{x}}_{tr}$ and $x_{r3}={\ddot{x}}_{tr}$ represent the respective reference motion states. $k_{11}$, $k_{12}$, $k_{13}$, $k_{21}$, $k_{22}$ and $k_{23}$ are controller parameters to be adjusted according to the trajectory tracking response.

Applying controllers Equations (18) and (19) to EHSA and EMA respectively leads to the motion state synchronization of EHSA and EMA, because all motion states of EHSA and EMA will asymptotically track the same reference trajectory $x_{r}={\left[x_{r1},\;x_{r2},\;x_{r3}\right]}^{T}$.

However, from the control laws Equations (18) and (19) for EHSA and EMA, the control laws require all the state variables of EHSA and EMA, i.e., displacements, velocities and accelerations. However, only the displacement sensors, which could measure the angular displacement of the control surface or the linear displacement of the cylinder, are available for the practical aviation actuation system on the aircrafts. Therefore, a specifically designed state observer is required to provide the requested immeasurable state variables.

To this end, the ESO will be designed for EHSA and EMA to observe system state variables along with the uncertainties in the next subsection.

### 3.2. LESO-Based Motion Synchronization Controller

#### 3.2.1. LESO Design

In this subsection, LESO will be designed for EHSA and EMA respectively, to estimate the uncertainties and the motion states of the actuation systems. The ESO regards all factors affecting the plant, including nonlinearities, uncertainties, and disturbances as a total uncertainty (i.e., extended state) which needed be observed [19,20,21]. The advantages for involving the extended state is its relatively independence of the mathematical model of the plant, better performance and simplification for implementing.

For EHSA system $\Psi_{\mathrm{EHSA}}$, let $b_{1}=b_{1n}+\Delta b_{1}$, assume $b_{1n}$ is the nominal value of $b_{1}$ and $\Delta b_{1}$ is the associated uncertainties, we have:

(20) $$b_{1}=b_{1n}+\Delta b_{1}$$

Then define the uncertainty needed to be estimated as:

(21) $$d_{1}=f_{1}(x_{1})+g_{1}+\varphi_{1}+\Delta b_{1}u_{1}$$

Let the extended state of ESO $x_{14}=d_{1}$, then EHSA system $\Psi_{\mathrm{EHSA}}$ could be extended and be rewritten as:

(22) $${\bar{\Psi }}_{\mathrm{EHSA}}:\;\{\begin{array}{c} {\dot{x}}_{11}=x_{12}\\ {\dot{x}}_{12}=x_{13}\\ {\dot{x}}_{13}=x_{14}+b_{1n}u_{1}\\ {\dot{x}}_{14}=h_{1}\\ \;y=x_{11} \end{array}$$

where $h_{1}={\dot{d}}_{1}$ is the changing rate of the uncertainty and it is assumed to be bounded.

The extended-order system ${\bar{\Psi }}_{\mathrm{EHSA}}$ can be rewritten by defining $X_{1}={\left[x_{11},\;x_{12},\;x_{13},\;x_{14}\right]}^{T}$ as the extended state vector:

(23) $$\{\begin{array}{c} {\dot{X}}_{1}={\bar{A}}_{1}X_{1}+{\bar{B}}_{1}u_{1}+{\bar{E}}_{1}h_{1}\\ \;y={\bar{C}}_{1}X_{1} \end{array}$$

where ${\bar{A}}_{1}=\left[\begin{array}{cccc} 0 & 1 & 0 & 0\\ 0 & 0 & 1 & 0\\ 0 & 0 & 0 & 1\\ 0 & 0 & 0 & 0 \end{array}\right]$, ${\bar{B}}_{1}=\left[\begin{array}{c} 0\\ 0\\ b_{1n}\\ 0 \end{array}\right]$, ${\bar{C}}_{1}={\left[\begin{array}{c} 1\\ 0\\ 0\\ 0 \end{array}\right]}^{T}$, ${\bar{E}}_{1}=\left[\begin{array}{c} 0\\ 0\\ 0\\ 1 \end{array}\right]$.

Now, we can design the ESO for ${\bar{\Psi }}_{\mathrm{EHSA}}$ as:

(24) $${\dot{\hat{X}}}_{1}={\bar{A}}_{1}{\hat{X}}_{1}+{\bar{B}}_{1}u_{1}+L_{1}{\bar{C}}_{1}\left(X_{1}-{\hat{X}}_{1}\right)$$

where ${\hat{X}}_{1}={\left[{\hat{x}}_{11},\;{\hat{x}}_{12},\;{\hat{x}}_{13},\;{\hat{x}}_{14}\right]}^{T}$ is the observed state vector of ESO, $L_{1}={\left[\beta_{11},\;\beta_{12},\;\beta_{13},\;\beta_{14}\right]}^{T}$ is the gain vector of the designed observer.

The ESO given in Equation (24) is a linear one, i.e., LESO, and it has several advantages. The structure of LESO is simple and is easy to be implemented on the practical servo system. Then, the observer gain $L_{1}$ can be solved systematically through pole placement and one typical example of $L_{1}$ is given as:

(25) $$L_{1}={\left[\beta_{11},\;\beta_{12},\;\beta_{13},\;\beta_{14}\right]}^{T}={\left[4\omega_{1},\;6\omega_{1}^{2},\;4\omega_{1}^{3},\;\omega_{1}^{4}\right]}^{T}$$

where $\omega_{1}>0$ is the only tuning parameter of the LESO, which could be thought as the bandwidth of the observer. Lastly, the closed-loop stability for LESO can be established conclusively, as shown in the next subsection.

Now we can use the same technique to design LESO EMA. Firstly, the EMA system $\Psi_{\mathrm{EMA}}$ is also extended as:

(26) $${\bar{\Psi }}_{\mathrm{EMA}}:\;\{\begin{array}{c} {\dot{x}}_{21}=x_{22}\\ {\dot{x}}_{22}=x_{23}\\ {\dot{x}}_{23}=x_{24}+b_{2n}u_{2}\\ {\dot{x}}_{24}=h_{2}\\ \;y=x_{21} \end{array}$$

where $x_{24}=d_{2}=f_{2}(x_{2})+g_{2}+\varphi_{2}+\Delta b_{2}u_{2}$ is the extended state of ${\bar{\Psi }}_{\mathrm{EMA}}$ which represents the uncertainty needs to be estimated, $\Delta b_{2}=b_{2}-b_{2n}$ is the uncertainty in input channel with $b_{2n}$ representing the nominal value of $b_{2}$, $h_{1}={\dot{d}}_{1}$ is the changing rate of the uncertainty.

Similar to EHSA, we can design the LESO for ${\bar{\Psi }}_{\mathrm{EMA}}$ as:

(27) $${\dot{\hat{X}}}_{2}={\bar{A}}_{2}{\hat{X}}_{2}+{\bar{B}}_{2}u_{2}+L_{2}{\bar{C}}_{2}\left(X_{2}-{\hat{X}}_{2}\right)$$

where $L_{2}={\left[4\omega_{2},\;6\omega_{2}^{2},\;4\omega_{2}^{3},\;\omega_{2}^{4}\right]}^{T}$, ($\omega_{2}>0$) is the observer gain vector, $X_{2}={\left[x_{21},\;x_{22},\;x_{23},\;x_{24}\right]}^{T}$, ${\bar{A}}_{2}={\bar{A}}_{1}$, ${\bar{B}}_{2}={\left[0,\;0,\;b_{2n},\;0\right]}^{T}$, ${\bar{C}}_{2}={\bar{C}}_{1}$.

#### 3.2.2. LESO-based Motion Synchronization Controller

In order to synchronize the motion states of EHSA and EMA, LESO-based motion synchronization controller will be designed to make sure that EHSA and EMA tracking the same reference trajectory $x_{r}={\left[x_{r1},\;x_{r2},\;x_{r3}\right]}^{T}$.

For EHSA, based on state feedback linearization controller Equations (14) and (18), the motion synchronization controller can be designed by utilized the state observation results of LESO Equation (24) as:

(28) $$u_{1}=\frac{1}{b_{1n}}\left[{\dot{x}}_{r3}+k_{11}(x_{r1}-{\hat{x}}_{11})+k_{21}(x_{r2}-{\hat{x}}_{12})+k_{31}(x_{r3}-{\hat{x}}_{13})-{\hat{x}}_{14}\right]$$

where ${\hat{x}}_{14}$ is the estimation of the uncertainty $d_{1}$ and is used to compensate the lumped effect of nonlinearities, uncertainties and disturbances in EHSA system.

With the controller Equation (28) and the observer Equation (24), the closed-loop stability of EHSA system $\Psi_{\mathrm{EHSA}}$ will be analyzed in the following.

Rewrite EHSA system $\Psi_{\mathrm{EHSA}}$ as:

(29) $${\dot{x}}_{1}=A_{1s}x_{1}+B_{1s}u_{1}+B_{1d}d_{1}$$

where $A_{1s}=\left[\begin{array}{ccc} 0 & 1 & 0\\ 0 & 0 & 1\\ 0 & 0 & 0 \end{array}\right]$, $B_{1s}=\left[\begin{array}{c} 0\\ 0\\ b_{1n} \end{array}\right]$,$B_{1d}=\left[\begin{array}{c} 0\\ 0\\ 1 \end{array}\right]$.

Noting that $x_{1}={\left[x_{11},\;x_{12},\;x_{13}\right]}^{T}$, ${\hat{x}}_{1}={\left[{\hat{x}}_{11},\;{\hat{x}}_{12},\;{\hat{x}}_{13}\right]}^{T}$ and $x_{r}={\left[x_{r1},\;x_{r2},\;x_{r3}\right]}^{T}$, the motion synchronization controller Equation (28) of EHSA can be rewritten as:

(30) $$u_{1}=K_{1s}x_{r}-K_{1s}{\hat{x}}_{1}+\frac{1}{b_{1n}}{\dot{x}}_{r3}-\frac{1}{b_{1n}}{\hat{x}}_{14}$$

where $K_{1s}=(1/b_{1n})[k_{11}\;k_{12}\;k_{13}]$ is the controller gain vector of EHSA system $\Psi_{\mathrm{EHSA}}$.

By using the same controller design technique, it is easy to design the motion synchronization controller for EMA base on state feedback linearization controller and LESO Equation (27), which is given as:

(31) $$u_{2}=\frac{1}{b_{2n}}\left[{\dot{x}}_{r3}+k_{21}(x_{r1}-{\hat{x}}_{21})+k_{22}(x_{r2}-{\hat{x}}_{22})+k_{23}(x_{r3}-{\hat{x}}_{23})-{\hat{x}}_{24}\right]$$

where ${\hat{x}}_{24}$ is the estimation of the uncertainty $d_{2}$ and is used to compensate unmodeled dynamics, model uncertainties and unknown external disturbances of EMA. Similarly, consider the control (30) and the observer (26), then the EMA system $\Psi_{\mathrm{EMA}}$ can be rewritten as:

(32) $${\dot{x}}_{2}=A_{2s}x_{2}+B_{2s}u_{2}+B_{2d}d_{2}$$

where $A_{2s}=\left[\begin{array}{ccc} 0 & 1 & 0\\ 0 & 0 & 1\\ 0 & 0 & 0 \end{array}\right]$, $B_{2s}=\left[\begin{array}{c} 0\\ 0\\ b_{2n} \end{array}\right]$, $B_{2d}=\left[\begin{array}{c} 0\\ 0\\ 1 \end{array}\right]$.

Then the motion synchronization controller (30) of EMA can be rewritten as:

(33) $$u_{2}=K_{2s}x_{r}-K_{2s}{\hat{x}}_{2}+\frac{1}{b_{2n}}{\dot{x}}_{r3}-\frac{1}{b_{2n}}{\hat{x}}_{14}$$

where $K_{2s}=(1/b_{2n})[k_{21}\;k_{22}\;k_{23}]$ is the controller gain vector of EHSA system $\Psi_{\mathrm{EMA}}$.

#### 3.2.3. Stability Analysis

The stability analysis of the closed-loop control system with the proposed LESO-based motion synchronization controllers will be discussed in this part.

Defining the state tracking error vector of $\Psi_{\mathrm{EHSA}}$ as:

(34) $$e_{1c}=x_{r}-x_{1}$$

Then its dynamics is given as:

(35) $${\dot{e}}_{1c}={\dot{x}}_{r}-{\dot{x}}_{1}$$

It is obviously that the following equation holds:

(36) $${\dot{x}}_{r}=A_{1S}x_{r}+B_{1d}{\dot{x}}_{r3}$$

Then, considering Equations (29), (30) and (36), the state tracking error dynamics can be expressed as:

(37) $${\dot{e}}_{1c}=\left(A_{1s}-B_{1s}K_{1s}\right)e_{1c}-\left[\begin{array}{cc} B_{1s}K_{1s} & B_{1d} \end{array}\right]e_{1o}$$

where $e_{1o}=X_{1}-{\hat{X}}_{1}$ is the observer estimation error vector of LESO Equations (24).

According to Equations (23) and (24), the observer error dynamics ${\dot{e}}_{1o}$ can be given as:

(38) $$\begin{array}{ccc} {\dot{e}}_{1o} & = & {\dot{X}}_{1}-{\dot{\hat{X}}}_{1}\\ & = & \left({\bar{A}}_{1}-L_{1}{\bar{C}}_{1}\right)e_{1o}+{\bar{E}}_{1}h_{1} \end{array}$$

Combining Equations (37) and (38) leads to:

(39) $$\left[\begin{array}{c} {\dot{e}}_{1c}\\ {\dot{e}}_{1o} \end{array}\right]=\left[\begin{array}{cc} \left(A_{1s}-B_{1s}K_{1s}\right) & -\left[\begin{array}{cc} B_{1s}K_{1s} & B_{1d} \end{array}\right]\\ 0 & \left({\bar{A}}_{1}-L_{1}{\bar{C}}_{1}\right) \end{array}\right]\left[\begin{array}{c} e_{1c}\\ e_{1o} \end{array}\right]+\left[\begin{array}{c} 0\\ {\bar{E}}_{1} \end{array}\right]h_{1}$$

The closed-loop stability of EHSA system $\Psi_{\mathrm{EHSA}}$ can be verified by checking the eigenvalues of the system matrix of the error dynamics Equation (39) which are determined by the eigenvalues of $\left(A_{1s}-B_{1s}K_{1s}\right)$ and $\left({\bar{A}}_{1}-L_{1}{\bar{C}}_{1}\right)$.

Since the pair $\left(A_{1s},\;B_{1s}\right)$ is controllable and the pair $\left({\bar{A}}_{1},\;{\bar{C}}_{1}\right)$ is observable, the stability of the error dynamics Equation (39) can always be ensured by placing the controller and observer poles appropriately. Furthermore, since the error dynamics Equation (39) is stable, it is obvious that, under the assumption of boundedness of $h_{1}$, the bounded-input–bounded-output stability for the dynamics Equation (39) is guaranteed. A Specially, when the changing rate $h_{1}$ of the uncertainty $d_{1}$ is reasonably small, the error dynamics Equation (39) is asymptotically stable.

With the LESO Equation (27) and the controller Equation (31), the closed-loop stability of $\Psi_{\mathrm{EMA}}$ can be studied and the error dynamics of the closed-loop EMA system can be given as:

(40) $$\left[\begin{array}{c} {\dot{e}}_{2c}\\ {\dot{e}}_{2o} \end{array}\right]=\left[\begin{array}{cc} \left(A_{2s}-B_{2s}K_{2s}\right) & -\left[\begin{array}{cc} B_{2s}K_{2s} & B_{2d} \end{array}\right]\\ 0 & \left({\bar{A}}_{2}-L_{2}{\bar{C}}_{2}\right) \end{array}\right]\left[\begin{array}{c} e_{2c}\\ e_{2o} \end{array}\right]+\left[\begin{array}{c} 0\\ {\bar{E}}_{2} \end{array}\right]h_{2}$$

where $e_{2c}=x_{r}-x_{2}$ is the state tracking error vector of $\Psi_{\mathrm{EMA}}$, $e_{2o}=X_{2}-{\hat{X}}_{2}$ is the observer estimation error vector of LESO Equation (27). Other variables or matrixes are defined similar to the relative ones in Equation (39).

Similar to the EHSA system, the same results about the stability of the closed-loop EMA system can be concluded.

## 4. Simulation Results and Discussion

### 4.1. Simulation Setup and Controller Parameters

In order to verify the advantage of the proposed LESO-based motion synchronization control algorithm, it is implemented on a simulation model of HAS of large civil aircraft. The simulation is established in MATLAB/Simulink environment and the parameters of the simulation model are listed in Table 1.

The parameters of the proposed motion synchronization controller are calculated as follows: $k_{11}=2000$, $k_{12}=8000$, $k_{13}=565$, $k_{21}=200$, $k_{22}=2446$, $k_{23}=30$ Then the parameter of LESO are set to $\omega_{1}=\omega_{2}=10,000$.

In addition, in order to illustrate the performance of LESO-based motion synchronization controller more objectively, the following three control algorithms will be compared in two simulation scenarios:

Controller C1: the classical PID control with parameters: EHSA: $K_{Ph}=3.8$, $K_{Ih}=20$, $K_{Dh}=0.1$; EMA: $K_{Pm}=16$, $K_{Im}=2$, $K_{Dm}=0.5$;
Controller C2: the state-difference feedback approach proposed by Cochoy et al. [4]. In this controller, the same PID control parameters with Controller C1 are used, and state-difference feedback coefficients are: $k_{SDF\_x}=150$, $k_{SDF\_v}=10$, $k_{SDF\_f}=2.0\times 10^{-6}$;
Controller C3: the proposed LESO-based motion synchronization controller.

### 4.2. Simulation Results with Step Signal Input Command

**Figure 3:**
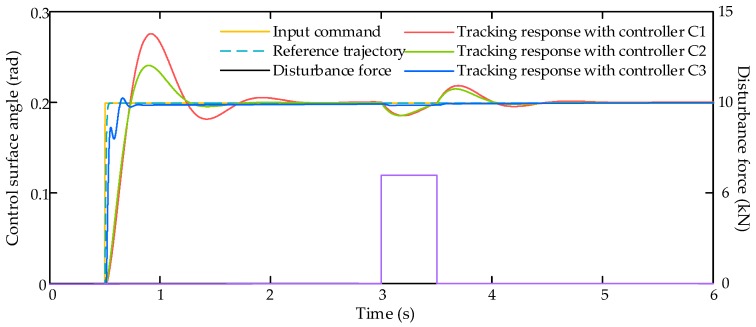
Tracking responses with step signal input command.

**Figure 4:**
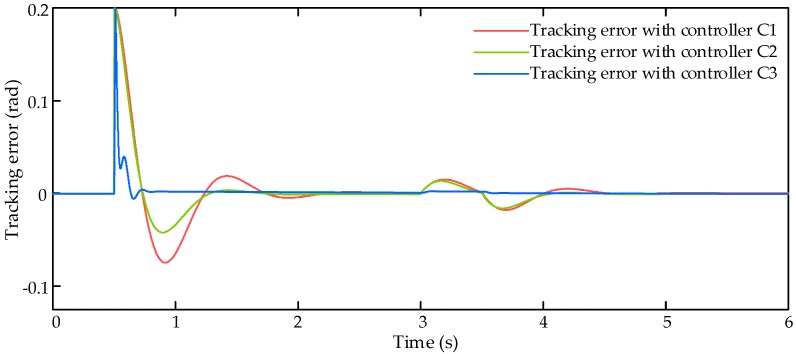
Tracking errors with step signal input command.

**Figure 5:**
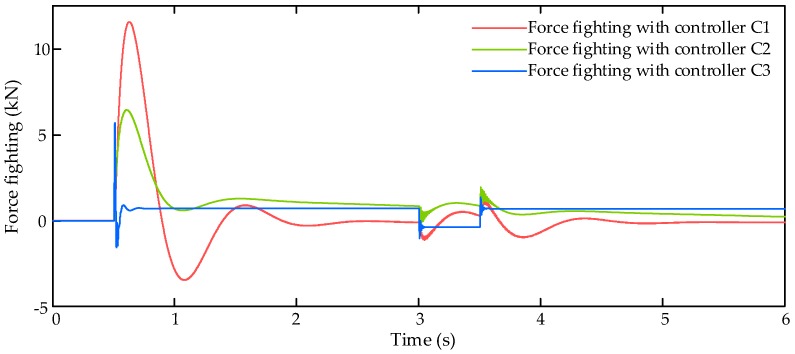
Force fighting results with step signal input command.

In this simulation scenario, the input command $x_{cmd}$ is set as a step signal with amplitude of 0.2 rad. In addition to the proportional aerodynamic load, an external air disturbance force $F_{air}$ with amplitude of 6000 N is added on the control surface to verify the robustness of the controllers. Then the simulation results are shown in Figure 3, Figure 4 and Figure 5.

The input command from flight control system and tracking responses with three compared controllers C1–C3 is shown in Figure 3, and corresponding tracking errors of these three controllers is shown in Figure 4. The comparative results in these two figures reveal that the proposed control algorithm achieve better dynamic tracking response and less tracking error than other two controllers.

The force fighting results of three compared controllers C1–C3 is shown in Figure 5, and it is evident that the proposed control algorithm can get better force fighting elimination than other two controllers, thanks to the well-designed LESO which gets all motion states of EHSA and EMA.

### 4.3. Simulation Results with Dynamic Signal Input Command

**Figure 6:**
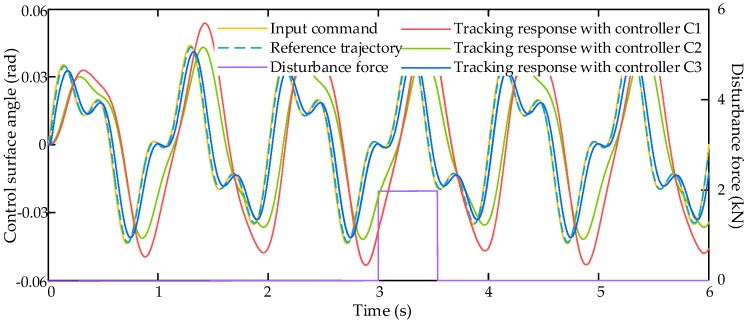
Tracking responses with dynamic signal input command.

**Figure 7:**
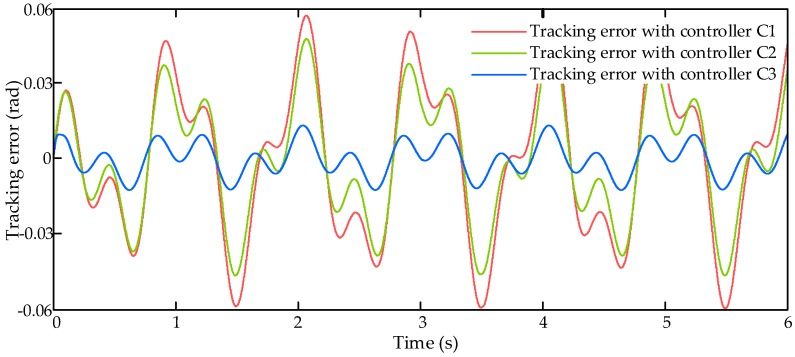
Tracking errors with dynamic signal input command.

**Figure 8:**
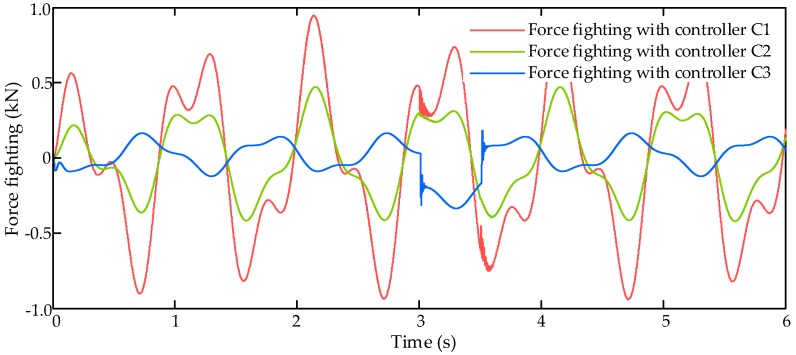
Force fighting results with dynamic signal input command.

The comparative simulation also runs with dynamic signal input command $x_{cmd}$ (dashed yellow line in Figure 6) and an external air disturbance force $F_{air}$ with amplitude of 2000 N (black line in Figure 6). Then the simulation results are shown in Figure 6, Figure 7 and Figure 8.

Figure 6 shows the tracking responses with three compared controllers C1–C3 along with the input command and the reference trajectory, and it indicates that the proposed LESO-based controller C3 has better tracking performance than other two. The tracking errors of these three controllers is shown in Figure 7. From Figure 7, we can find that the tracking error under controller C1 is relatively large, reaching to 0.06 rad. The tracking error under controller C2 is better than C1, the maximum tracking error is about 0.05 rad, but it is still unsatisfactory. The proposed controller C3 can achieve the best tracking performance among these three controllers, and its maximum tracking error is about 0.013 rad. It illustrates that the proposed LESO-based motion synchronization controller can improve the dynamic tracking performance of the HAS.

Figure 8 shows the force fighting results with dynamic signal input command under three controllers C1–C3. From Figure 8, we can find that the proposed LESO-based controller C3 has the best force fighting reduction performance in these three controllers and the force difference between EHSA and EMA can be reduced to 0.15 kN in normal condition and 0.34 kN under disturbance.

### 4.4. Simulation Results with Real Flight Control Command under Random Perturbance

**Figure 9:**
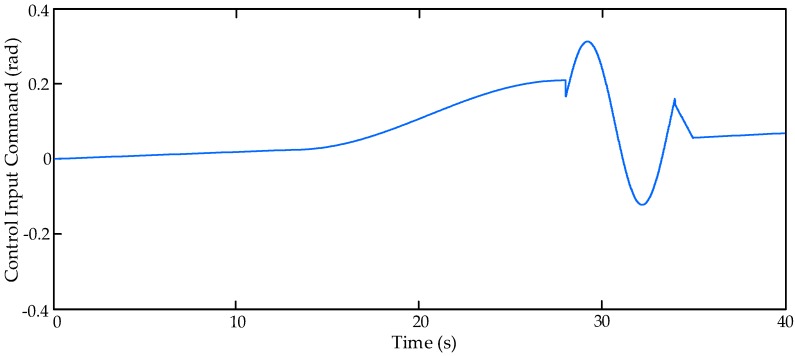
Flight control input command of the hybrid actuation system (HAS).

In order to verify the control performance of the proposed controller under unknown disturbance, the comparative simulation runs with real flight control command under random perturbance. In this simulation scenario, the input command of the HAS is set as a piece of real flight control command, which is shown in Figure 9.

**Figure 10:**
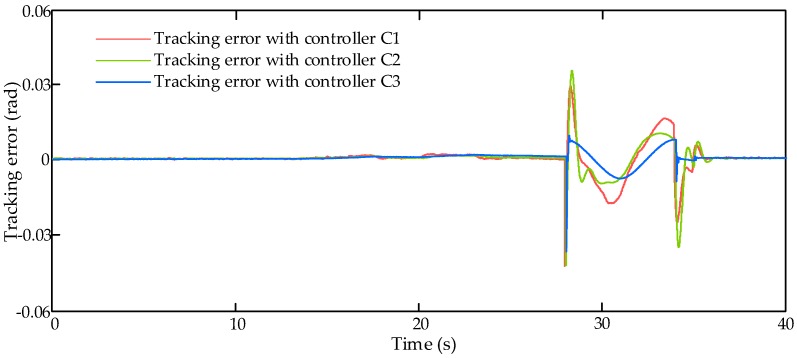
Tracking errors with real flight control command under random perturbance.

**Figure 11:**
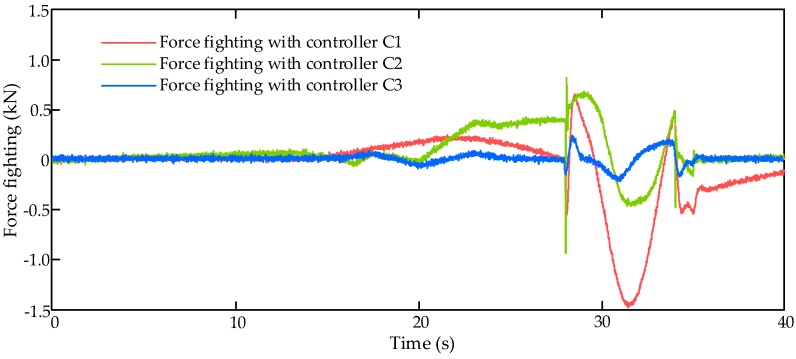
Force fighting results with real flight control command under random perturbance.

In this simulation, the external air disturbance force is set as a proportional load, i.e., $F_{air}=k\cdot \theta_{cs}$, here $k=3.75\times 10^{2}$ Nm/rad. In addition, a random perturbance of aerodynamic load within the range of ± 50 Nm is added on the control surface. Then the comparative simulation results of these three controllers is given in Figure 10 and Figure 11.

Figure 10 shows the tracking errors with three compared controllers, C1, C2 and C3, with real flight control command under random perturbance. From Figure 10, it can be found that all of these three controllers can obtain good tracking performance when the flight control command changes slowly (0 s–27 s), however, when the flight control command changes quickly (27 s–40 s), the control performance of the proposed controller C3 is much better than other two controllers. The better dynamic performance of the actuation system can lead to the improvement of the aircraft flight performance.

Figure 11 shows the force fighting results with three compared controllers with real flight control command under random perturbance. It is clear that the proposed LESO-based controller C3 can significantly eliminate the force difference (Blue line) between EHSA and EMA.

This simulation indicates that the proposed LESO-based controller can achieve good dynamic command tracking performance under nonlinear and unknown perturbance.

## 5. Conclusions

LESO-based motion synchronization control strategy has been proposed in this paper for hybrid actuation system of more electric aircraft. All motion states of EHSA and EMA can be observed by the well-designed LESO and the motion synchronization controller can ensure EHSA and EMA to simultaneously track the desired motion trajectory from flight control system. As a result, the force fighting can be greatly reduced due to synchronous motion of EHSA and EMA. The robustness of the closed-loop system is guaranteed by introducing the forth state of LESO, because the forth state of LESO can estimate and compensate the lumped effect of nonlinearities, uncertainties and unknown disturbances. Finally, the effectiveness of the proposed LESO-based control strategy is validated by comparative simulation results under different conditions.

## Figures and Tables

**Table 1:** The parameters of hybrid actuation system.

Main Parts in HAS	Parameters	Values	Units
Electro-hydraulic servo actuator (EHSA)	Amplification coefficient of servo valve. $K_{v}$	3.04 × 10^−4^	m/A
	Flow/opening gain $K_{q}$	2.7	m^2^/s
	Flow/pressure gain $K_{c}$	1.75 × 10^−11^	(m^3^/s)/Pa
	Piston area of EHSA cylinder $A_{h}$	1.1 × 10^−3^	m^2^
	Volume of the piston chamber $V_{h}$	1.1 × 10^−4^	m^3^
	mass of piston and rod $m_{h}$	25	Kg
	Damping coefficient $B_{h}$	1 × 10^4^	N·s/m
	Effective bulk modulus $E_{h}$	8.0 × 10^8^	Pa
	Total leakage coefficient $C_{hl}$	1.0 × 10^−11^	(m^3^/s)/Pa
Electromechanical actuator (EMA)	Back-EMF coefficient $K_{e}$	0.161	V/(rad/s)
	Inductance of motor $L_{e}$	4.13 × 10^−3^	H
	Resistance of motor $R_{e}$	0.54	Ω
	Electromagnetic coefficient $K_{em}$	0.64	Nm/A
	Total moment of inertia of rotating parts $J_{m}$	1.136 × 10^−3^	Kg·m^2^
	Damping coefficient $B_{m}$	4.0 × 10^−3^	Nm·s/rad
	Transmission coefficient $K_{gs}$	1.256 × 10^3^	rad/m
	Transmission efficiency η	0.9	
Control Surface	Connection stiffness of EHSA $K_{h}$	1 × 10^8^	N/m
	Connection stiffness of EMA $K_{m}$	1 × 10^8^	N/m
	Radial distance of control surface $r_{cs}$	0.1	m
	Moment of inertia of control surface ($J_{cs}$)	6.0	Kg·m^2^
